# Supramolecular Loading of DNA Hydrogels with Dye–Drug Conjugates for Real‐Time Photoacoustic Monitoring of Chemotherapy

**DOI:** 10.1002/advs.202204330

**Published:** 2022-11-20

**Authors:** Raina M. Borum, Colman Moore, Yash Mantri, Ming Xu, Jesse V. Jokerst

**Affiliations:** ^1^ Department of NanoEngineering University of California, San Diego 9500 Gilman Drive La Jolla California 92093 United States; ^2^ Department of BioEngineering University of California, San Diego 9500 Gilman Drive La Jolla California 92093 United States; ^3^ Department of Radiology University of California, San Diego 9500 Gilman Drive La Jolla California 92093 United States; ^4^ Materials Science Program University of California, San Diego 9500 Gilman Drive La Jolla California 92093 United States

**Keywords:** bioimaging, drug delivery, drug monitoring, hydrogel, image‐guided therapy, in vivo molecular imaging, photoacoustic imaging

## Abstract

A longstanding problem with conventional cancer therapy is the nonspecific distribution of chemotherapeutics. Monitoring drug release in vivo via noninvasive bioimaging can thus have value, but it is difficult to distinguish loaded from released drug in live tissue. Here, this work describes an injectable supramolecular hydrogel that allows slow and trackable release of doxorubicin (Dox) via photoacoustic (PA) tomography. Dox is covalently linked with photoacoustic methylene blue (MB) to monitor Dox before, during, and after release from the hydrogel carrier. The conjugate (MB‐Dox) possesses an IC50 of 161.4 × 10^−9^ m against human ovarian carcinoma (SKOV3) cells and loads into a DNA‐clad hydrogel with 91.3% loading efficiency due to MB‐Dox's inherent intramolecular affinity to DNA. The hydrogel is biodegradable by nuclease digestion, which causes gradual release of MB‐Dox. This release rate is tunable based on the wt% of the hydrogel. This hydrogel maintains distinct PA contrast on the order of days when injected in vivo and demonstrates activatable PA spectral shifts   during hydrogel degradation. The released and loaded payload can be imaged relative to live tissue via PA and ultrasound signal being overlaid in real‐time. The hydrogel slowed the rate of the murine intraperitoneal tumor growth 72.2% more than free Dox.

## Graphical abstract

This report introduces a multifunctional hydrogel that allows (1) real‐time surveillance of drug release via photoacoustic tomography overlaid with ultrasound and (2) slow and local delivery of chemotherapy from gradual hydrogel degradation. The hydrogel is composed of cross‐linked DNA strands that serve as the scaffold. Doxorubicin (Dox) is covalently tagged with methylene blue (MB) for photoacoustic imaging (PAI) contrast.

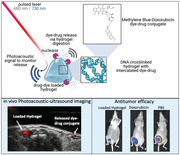

## 1 Introduction

Drug tracking in vivo offers value on understanding the therapeutic efficacy and pharmacokinetics of chemotherapeutics. For example, traditional cancer drugs can have nonspecific cytotoxicity. Emerging small molecule therapeutics may have different protein‐binding interactions, novel biodistribution pathways, downstream asynthetic phase I and II reactions (e.g., oxidation, reduction, methylation), unforeseen conjugation reactions during metabolism, or differences in excretion or clearance pathways.^[^ ^1^ ^]^ If molecular drugs can be monitored in detail and in real‐time, then their side effects and potencies—or lack thereof—can be better understood for improved strategies or personalized treatments against chronic diseases.

Biomedical imaging is a robust and multifunctional modality that can track drugs; unlike analyte detection in biological sampling, biomedical imaging allows both real‐time and spatial information on drug location.^[^ ^2^, ^3^ ^]^ A popular strategy to accomplish this is by combining the drug with a contrast agent when administered in vivo. However, several accessory capabilities from this pairing are essential for an image‐based drug tracking system to be considered viable: (1) quantitative drug monitoring^[^ ^3^ ^]^ or the ability to characterize the amount of drug present, loaded, or released in vivo; (2) activatable contrast change^[^ ^4^ ^]^ to report if the drug is released from a carrier, metabolized, or bound to other macromolecules; (3) sustained contrast from the drug for longitudinal monitoring; and (4) real‐time imaging in live tissue. There are many imaging modalities to use, but photoacoustic imaging (PAI) has gained particular traction in this context.^[^ ^5^, ^6^ ^]^ PAI is an ideal modality to monitor drugs in vivo because the incident near infrared (NIR) light can safely penetrate tissue to activate nano‐ and molecular‐scale contrast agents for acoustic broadband signal while simultaneously imaging live tissue with conventional ultrasound.^[^ ^7^, ^8^ ^]^ Unlike other modalities such as magnetic resonance imaging (MRI), computed tomography (CT), or radiography, PAI does not require full body scans or ionizing radiation.

Many strategies have demonstrated novel drug monitoring systems via PAI, but none satisfy all four abovementioned metrics. For example, Duan et. al. combined doxorubicin and NIR squaraine reporting dye into pH‐sensitive polymeric micelles for controlled release in the tumor microenvironment.^[^ ^9^ ^]^ Sun et al. later introduced a multifunctional nanoparticle that carries both chemotherapeutic Pt(II) metallacycle molecules and an NIR‐II molecular dye into melanin nanoparticles for combined imaging, chemotherapy, and photothermal therapy.^[^ ^10^ ^]^ However, the co‐loaded nature of the drug and the dye only provides image contrast with respect to the carrier alone or signal loss when the payload is released from the carrier. These systems do not enable contrast to follow the drug upon its release. This leads to crucial information on the overall pharmacokinetics of the drug itself.^[^ ^5^ ^]^ Jeevaranthiam and Lemaster et al. covalently linked a chemotherapy drug (paclitaxel) to the contrast agent (methylene blue, MB) for more precise drug localization during PAI. This new molecule remained invisible to PAI until its release from polymeric PLGA nanoparticles due to the changed oxidation of MB via the “blue bottle effect.”^[^ ^11^ ^]^ One limitation of this approach was that paclitaxel could only be imaged when released.

**Figure 1:**
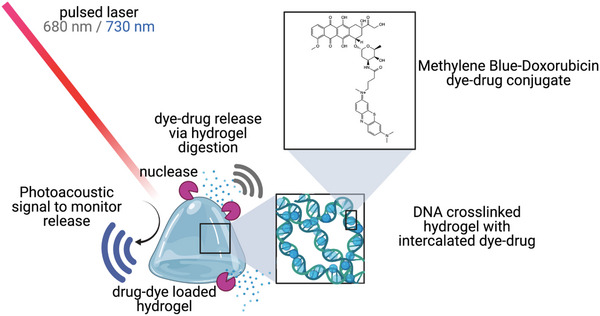
A biodegradable hydrogel photoacoustically monitors chemotherapeutic drug release. Here, a pure DNA cross‐linked hydrogel is loaded with methylene blue (MB) doxorubicin “MB‐Dox” dye–drug conjugate via hydrophobic binding. The MB‐Dox compound has activatable wavelength‐specific photoacoustic (PA) signal when loaded in the hydrogel and during drug release from photoacoustic signal from MB. The hydrogel degrades from in vivo nuclease activity.

Here, we describe a system where Dox is photoacoustically visible both when encapsulated and when released in vivo. Dox was first conjugated to MB for photoacoustic signal. This conjugate is then loaded into an injectable and biodegradable DNA cross‐linked hydrogel due to the molecule's strong intermolecular affinity to bind into double helical DNA with its planar and aromatic structure, and the facile ability to make DNA hydrogels with tunable mechanical strength and biodegradability.^[^ ^12^ ^]^ The tight packaging and heightened local concentration of the conjugate in the DNA hydrogel causes a redshift in the MB PA signal relative to conjugate free in solution phase. As a result, when the hydrogel gradually degrades from nuclease activity, local concentration gradients cause the released conjugate to produce a PA signal at a different wavelength. Thus, imaging can determine if the drug is loaded or released (**Figure** **1**). Furthermore, the gradual degradation of the hydrogel enables slow and controllable release of the payload, which in turn offers good antitumor efficacy relative to conventional chemotherapy.

## 2 Results and Discussion

### 2.1 Methylene Blue‐Doxorubicin Conjugate

#### 2.1.1 Chemical synthesis and characterization of “MB‐Dox”

**Figure 2:**
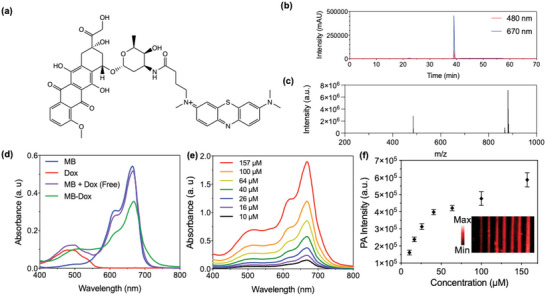
Chemical, optical, and photoacoustic characterization of MB‐Dox. a) Chemical structure of the conjugate—the reaction involves the amine group on the sugar moiety of doxorubicin and the NHS‐ester of NHS‐activated methylene blue (MB). b) Liquid chromatograms of purified MB‐Dox via HPLC where 480 nm absorbance and 670 nm absorbance are for Dox and MB, respectively, were monitored. c) Electrospray ionization (ESI) *m*/*z* for MB‐Dox C_46_H_49_N_4_O_12_S^+^, expected: 881.31; found: 881.3064. d) Optical absorbance spectra of MB, Dox, MB mixed with Dox unconjugated, and the MB‐Dox conjugate show the conjugate maintains curves from MB and Dox with a peak absorbance near 670 nm in water. e) Absorbance spectra of MB‐Dox with increasing micromolar concentrations in water show increasing absorbance at 670 nm with no MB mediated dimerization. f) PA image of tubes and quantified PA intensity show increasing concentration of MB‐Dox leads to increasing PA signal at 690 nm. The inset is a longitudinal image of the tubes loaded with respective increasing concentrations of MB‐Dox from left to right.

MB was chosen as the contrast agent due to its widespread use in medicine, long half‐life on the order of 5–6 h,^[^ ^13^, ^14^ ^]^ NIR excitation for photoacoustic (PA) signal, potential to link with Dox, and propensity to intercalate into DNA and thus a DNA‐constituted hydrogel.^[^ ^15^ ^]^ MB and Dox are Food and Drug Administration (FDA) approved drugs and have been used in the clinic for decades, but their co‐administration poses a unique advantage: Past reports have demonstrated that the redox potential of MB can overcome the cardiotoxic reactive oxygen generation from Dox without compromising antitumor efficacy.^[^ ^16^, ^17^ ^]^ MB was covalently linked to Dox (MB‐Dox) through a simple NHS‐ester‐amine reaction as outlined in (**Figure** **2**a and Figure S1, Supporting Information). The amine group on the sugar moiety of Dox allows it to react with an NHS‐ester attached to MB. This process was assisted by triethylamine (TEA) in DMSO solvent. After incubation, DMSO was removed from the reaction, and the sample was then purified via isocratic reverse phase high performance liquid chromatography (RP‐HPLC) in 30% acetonitrile solvent (Figure 2b and Figure S2, Supporting Information). The structure of this compound was confirmed via electrospray ionization mass spectrometry (ESI‐MS). The positive mode of ESI‐MS validated the characteristic 881.3 *m/z* ratio peak, which corresponded to the total molecular weight of the compound (Figure 2c). The compound was further characterized using ^1^H NMR (Figure S3, Supporting Information). The synthesis yield of this reaction was 14.8%.

#### 2.1.2 Optical MB‐Dox Characterization

The solubility of the compound was also observed from optical absorbance curves of the compound both in DMSO and in pure water (Figure 2d and Figure S4a,b, Supporting Information). The absorbance curve of the compound was representative of the additive curves found in MB and Dox on their own with a slight 3 nm red‐shifted peak from MB (Figure 2d and Figure S4a, Supporting Information). This confirmed that the molecule should still be viable for PAI due to this NIR optical peak absorption. Interestingly, increasing micromolar concentrations of the compound did not lead to increased or emergent secondary blue‐shifted peak formations commonly observed^[^ ^18^ ^]^ and as we demonstrated with MB alone, (Figure 2e and Figure S5a–c, Supporting Information), thus implying that the Dox appendage in the compound has a structural protective effect against typical MB dimerization in water. There is a need for molecular drugs to avoid uncontrollable aggregation in vivo,^[^ ^19^ ^]^ and thus this feature is advantageous for MB‐Dox's therapeutic viability.

#### 2.1.3 MB‐Dox Photoacoustic Characterization

**Figure 3:**
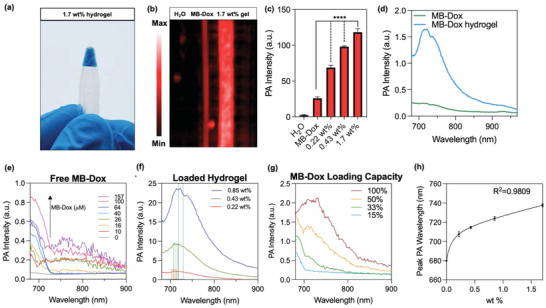
DNA hydrogel loaded with MB‐Dox. a) Photograph of cross‐linked 1.7 wt% DNA loaded with blue MB‐Dox in an upside‐down sample tube shows a highly loaded hydrogel. b) PA image of tubes comparing the PA signal enhancement from the loaded hydrogel in comparison to the PA signal of the same concentration of MB‐Dox free in water shows heightened contrast by the hydrogel. c) PA signal quantification demonstrates that increased wt% of hydrogel leads to increased PA signal when loaded with equal amounts of MB‐Dox. d) PA spectra of MB‐Dox hydrogel shows a peak near 730 nm while free MB‐Dox has peak PA intensity closer to 680 nm. e) It can be observed that with increasing concentrations of MB‐Dox in water, there is no wavelength shift in PA maximum absorbance (i.e., MB‐Dox maintains a PA peak at 680 nm). f) Increasing wt% of the MB‐Dox loaded hydrogel, on the other hand, not only shows an overall increase in PA intensity, but also an increasing red‐shifted PA peak from between 710 to 730 nm from 0.22 to 0.85 wt%, respectively. g) Spectral measurements on the hydrogel with changed amount of loaded MB‐Dox in terms of achieved loading capacity corroborates that the spectral shifts in panel (f) are in fact true to changing gel wt% as opposed to changing amount of added payload. h) PA spectral shifts are indicative of gel wt%.

The conjugate was then characterized for PA signal generation; 680 nm incident light was chosen for PA activation because it was the closest wavelength to MB‐Dox's peak optical absorbance curve. The PA signal increased with respect to increasing MB‐Dox concentration (Figure 2f), and the limit of detection (LoD) for this compound was found to be at 11.6 × 10^−6^ m. When the compound was irradiated with pulsed NIR light ranging from 680 to 900 nm, the photoacoustic intensity reached its maximum at 680 nm, which is characteristically seen in MB photoacoustic studies (**Figure** **3**e).^[^ ^11^ ^]^ Therefore, the free compound maintains a photoacoustic profile that is relatable to MB, and Dox does not affect this peak or compromise PA signaling.

#### 2.1.4 MB‐Dox Cytotoxicity

The compound's cytotoxicity was then tested and compared to that of MB and Dox at matching concentrations. The IC50 value of each molecule was evaluated with SKOV3. Dox alone maintained the lowest IC50 value (68.56 × 10^−9^ m), while MB‐Dox yielded an IC50 of 161.4 × 10^−9^ m, and MB of 1460 × 10^−9^ m (Figure S6a, Supporting Information). The IC50 for Dox reported here is similar to the 68 × 10^−9^ m IC50 of paclitaxel in Jeevaranthiam and Lemaster et al.’s work, however, their conjugate had an IC50 value that increased to 447 × 10^−9^ m.^[^ ^11^ ^]^

MB has also demonstrated photodynamic therapeutic effects upon NIR light irradiation.^[^ ^20^ ^]^ Therefore, we further exposed the cells incubated with the compound to a 100 mW 660 nm laser for 10 min, which was suitable for MB activation, and compared the cytotoxicity results to those left in the dark. The cytotoxicity increased by 37% when administered at the IC50 concentration and irradiated with light, but the overall photodynamic therapy (PDT) effects from the compound did not lead to significant cytotoxicity enhancement (*p* = 0.114) (Figure S6b, Supporting Information).

### 2.2 MB‐Dox Loaded Hydrogels

#### 2.2.1 Hydrogel Formulation and MB‐Dox Loading

Engineered nanoparticle systems have faced several challenges as carriers for small molecule drugs and chemotherapy.^[^ ^21^ ^]^ For example, some nanoparticle carriers experience premature burst effects, leaking, or degradation from lymphatic channels, the liver, urinary tract, or bile. On the other hand, other nanoparticle carriers are too stable for effective drug release while in the tumor microenvironment.^[^ ^22^ ^]^

A hydrogel was chosen as the MB‐Dox carrier for structural and functional reasons. First, hydrogels can load higher amounts of payload relative to nanoparticles, but their tunable mechanical strength can also lead to a tunable release rate of the payload.^[^ ^23^ ^]^ We thus hypothesized the payload may release slower than it were to be loaded into nanoparticles, which is ideal for systematic chemotherapeutic dosage against drug resistance.^[^ ^24^, ^25^ ^]^ Second, we hypothesized that a higher loading of cargo would increase local concentrations of the contrast agent and thus increase the signal‐to‐background ratio (SBR) in vivo. Higher PA contrast becomes increasingly essential as NIR background signal is ever‐present in tissue, blood, hemoglobin, melanin, and other endogenous absorbers.^[^ ^26^ ^]^

The macroscopic hydrogel in this work is composed of highly concentrated DNA strands cross‐linked by canonical base pairing rules. The rationale for DNA as the hydrogel skeleton is twofold. First, nucleic acids are a biocompatible and biodegradable material, and thus the hydrogel is inert to tissue while its degradation is inevitable due to ubiquitous nuclease activity. Second, small and planar aromatic molecules have strong affinity to the interior of DNA due to hydrophobic stacking interactions.^[^ ^27^ ^]^ MB is a common stain for nucleic acid while Dox's cytotoxic nature is due to its damage in cellular nucleic acid through intercalation.^[^ ^15^, ^28^ ^]^ In fact, DNA hydrogels and structures have widely demonstrated promise in harboring Dox molecules for drug delivery.^[^ ^28^, ^29^, ^30^ ^]^ Moreover, DNA nanostructures have been reported as viable scaffolds for photoacoustic contrast agents with the ability to even modulate their activatable wavelengths by controlling distances between discrete dye molecules.^[^ ^31^, ^32^, ^33^ ^]^ Besides the specific case of DNA hydrogels, other similarly aromatic driven stacking interactions have led to drug delivery systems with efficient loading capacity.^[^ ^34^ ^]^ Therefore, the MB‐Dox conjugate was expected to load strongly into a DNA‐based hydrogel.

The hydrogel in this work directly used Wang et. al.’s gelation design, which is cross‐linked by two amplifying single strand species and one gel‐forming initiator strand via the hybridization chain reaction (HCR).^[^ ^35^ ^]^ In brief, when the initiator strand binds to one amplifying strand through base‐pair specificity, that strand will bind with the other amplifying strand to form a cascaded self‐assembly of a hierarchical double‐stranded DNA superstructure. The highly concentrated DNA strands are linked by base pairing to form a superstructural gel after hybridization, and gel wt% tunability is possible by modulating the total DNA concentration in solution.

After the gel was synthesized, it was mixed gently with MB‐Dox. The drug loading efficiency (DLE) of MB‐Dox into the gel averaged 91.3% while the hydrogel loading content (DLC, i.e., loading content) was 52.4%; while most nanoparticle drug carrier systems are only starting to accomplish drug loading content over 10%, fabrication of these carriers is more labor intensive and loading efficiency can also suffer.^[^ ^36^ ^]^ These results therefore demonstrate the DNA hydrogel's competitive and robust drug loading potential. The hydrogel could load the compound on the order of hundreds of micromolar; it adopted MB‐Dox's blue color (Figure 3a and Figure S8, Supporting Information).

#### 2.2.2 Hydrogel PA Characterization

When irradiated with 680‐nm pulsed NIR laser, the photoacoustic signal from the loaded 1.7 wt% hydrogel was fivefold higher than the signal of same concentration of free MB‐Dox in water (Figure 3b–d), which can be explained by the forced aggregation between the molecules and a net decrease in efficient heat transfer between them.^[^ ^37^ ^]^ When the PA spectrum of the hydrogel was compared to the spectrum of the free MB‐Dox, however, the hydrogel showed a red‐shifted photoacoustic peak that ranged between 710 and 730 nm depending on the wt% of the hydrogel (Figure 3f). This characteristic energy shift is most likely due to the increased local concentration between the MB‐Dox molecules. The tight assembly of intercalated organic molecules into DNA resulted in increased J‐aggregation (“head to tail” molecular assembly), which characteristically red‐shifts peak absorption.^[^ ^38^, ^39^, ^40^ ^]^ The red‐shifted peak in the loaded hydrogel—both by increased weight % and increased loading capacity—confirm that the MB‐Dox molecule orients itself within double helical DNA via head‐to‐tail assembly rather than stacking. This change in peak wavelength is a valuable feature^[^ ^41^ ^]^ that can photoacoustically differentiate loaded MB‐Dox from free MB‐Dox in solution.

Patterns in PA spectral shifts can be exploited for quantitative characterization. Changes in gel wt% (i.e., fixing the added MB‐Dox amount in the gels while tuning their wt% in the solvent) resulted in signature PA wavelength shifts; thus, it was essential to investigate any characteristic shifts based on the extent of drug content. Therefore, PA spectra were plotted with respect to drug loading capacity in the hydrogels. When the hydrogels were loaded at up to 100% loading capacity (i.e., full drug loading content is achieved when loading the hydrogel), the PA peak remained at around 730 nm. The PA peak was at 680 nm below this amount, even at as much as 50% loading capacity, similar to free MB‐Dox in solution (Figure 3g). Drug loading capacity does not possess the same spectral shift behavior as is seen with the gel wt%. This can be explained by the fact that the MB‐Dox molecule is not intercalated within the hydrogel at full capacity: J‐aggregation is less prevalent with less red‐shifted PA wavelengths as observed at higher loading capacities. This behavior provides an advantage because spectral shifts from the hydrogel can be directly tied to change in wt% of the hydrogel itself, with no ambiguity as to whether the changed amount of loaded drug contributed to the shift. Therefore, spectral shifts of the hydrogel after in vivo administration can indicate the change in the gel wt% that can amount to quantitative capabilities when monitoring the hydrogel in live tissue (Figure 3h).

The photostability of the hydrogel was also evaluated. In brief, the hydrogels were exposed to the pulsed NIR laser for 15 min, and the change in PA intensity was monitored and analyzed. When hydrogels with different loaded amounts of MB‐Dox were tested, the PA intensity decreased below 90% at 33% loading capacity (Figure S9a,b, Supporting Information). On the other hand, when gels at 100% loading capacity but with different wt% ranging from 0.22 to 0.88 wt% were tested, the lowest photostability (for the 0.22 wt% hydrogel) still maintained 93% PA intensity (Figure S9d,e, Supporting Information). When free MB‐Dox was tested for photostability, the lowest concentration (17.53 × 10^−6^ m) had decreased photoacoustic intensity to 94% (Figure S9c,f, Supporting Information). These results underline the robust photostability of the compound and the loaded hydrogel.

#### 2.2.3 Hydrogel Degradation and Cytotoxicity

**Figure 4:**
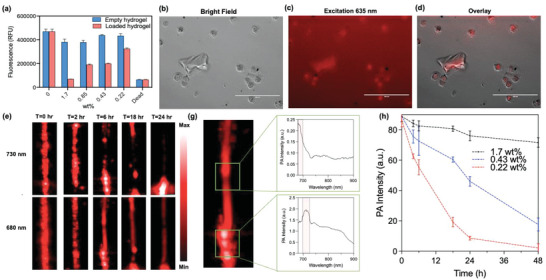
Hydrogel degradation and MB‐Dox release in vitro. a) Cytotoxicity measurements of SKOV3 cells with empty versus loaded hydrogels at differing wt% show that increasing wt% of loaded hydrogel is increasingly toxic to the cells while empty DNA hydrogels had no toxicity. b) Bright field image of dying SKOV3 cells and hydrogel fragments. c) Fluorescent image with excitation of 635 nm of the same image and d) overlay show fluorescence from the MB is present in both the hydrogel fragment and dying cells and that the MB‐Dox in fact enters inside the cells for its cytotoxic effects. e) PA images of degrading MB‐Dox loaded hydrogel sample at different wavelengths show a decrease in PA signal at 730 nm while PA signal at 680 nm becomes more uniform, thus outlining the decay of the hydrogel and release of free MB‐Dox. g) Spectral scanning at different areas of the reaction tube elucidate where the degrading hydrogel fragments are versus released free MB‐Dox by their characteristic 730 and 680 nm PA peaks, respectively. h) Decreased PA intensity of the hydrogels at 730 nm with different wt% DNA hydrogels indicate that decreasing wt% leads to faster degradation and faster decrease in hydrogel PA signal when scanned at time points over 48 h (*n* = 3).

The transient nature of the hydrogel is due to its digestion from rich nuclease activity in biological fluids. When the hydrogel was administered to SKOV3 cell cultures, its initial visibility to the naked eye disappeared over the span of 24–48 h depending on the wt% of the gel. Yet because MB is also a fluorescent molecule, fragments of the hydrogel and the payload with respect to the cells could be monitored using fluorescent cell microscopy: A fluorescent channel with an excitation wavelength of 635 nm was sufficient to localize MB signal. After 24‐h hydrogel incubation, cell death was apparent while micron‐ranged gel fragments were present in the media. Red fluorescent signal was seen both from unreleased MB‐Dox inside the fragments, and inside of the dying cells (**Figure** **4**b–d). It was therefore corroborated that the cytotoxic effects of the hydrogel against the cells (Figure 4a) was due to MB‐Dox release and internalization. MB‐Dox was also incubated in cell culture media for 24 h at 37 °C and characterized via ESI‐MS; it was confirmed that the compound maintained molecular integrity and was not cleaved by biological media (Figure S7, Supporting Information).

The release kinetics of the transient hydrogel was monitored with respect to PAI. The hydrogel was incubated in 100% mouse serum at 37 °C, and the same sample was imaged over the span of 48 h. Samples of different wt% were evaluated. The hydrogel‐serum samples were confined in plastic tubing and were not removed or agitated during the entire experiment. The tubes initially showed strong PA signal throughout the tube, but the signal became less uniformly distributed by 6 h of incubation time, and fragmentation of the gel with free floating or released MB Dox became increasingly apparent (Figure 4g). Release kinetics was quantified using optical absorbance measurements alongside PA signal with independent samples: The increased absorbance of MB‐Dox was monitored by measuring the supernatant surrounding the degrading hydrogel, while the decreased absorbance of DNA was monitored at the sediment at the bottom of the reaction tube (Figure S10, Supporting Information).

However, during the PA monitoring of hydrogel degradation, the same sample was imaged both at 730 and 680 nm wavelengths, which are the peak PA wavelengths for the hydrogel and free MB‐Dox, respectively. Over time, the PA signal across the tube at 730 nm scanning decreased and became less uniform while the PA signal at 680 nm became increasingly uniform throughout the entirety of the tube (Figure 4e). Spectral measurements were then taken at locations in the tube were there was more concentrated PA signal observed in the images, and the peak at these regions corresponded to the hydrogel spectra. These measurements illustrated that multiwavelength scanning could clarify both the structural decay of the hydrogel and the release of the MB‐Dox that spread throughout the tube.

### 2.3 Hydrogel In Vivo Performance

#### 2.3.1 Hydrogel Photoacoustic Evaluation in Live Tissue

**Figure 5:**
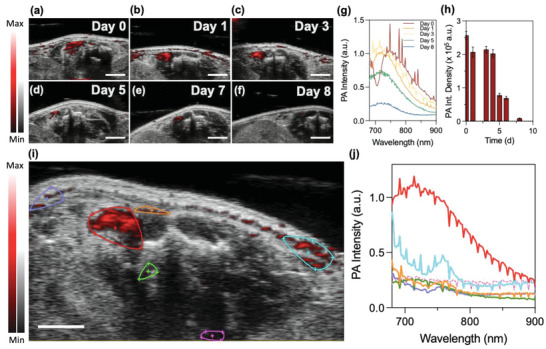
Hydrogel in vivo photoacoustic imaging (PAI). a–f) In vivo B‐mode and PA overlay images show that the hydrogel maintains strong contrast under the same PA gain and remains in the same location after it was injected into a mouse over the span of 8 days. g) Corresponding PA spectra of the hydrogel over 8 days shows a gradual blue‐shifted PA peak from 750 to 710 nm due to decreased wt% of the hydrogel from nuclease digestion in vivo. h) A decrease in in vivo PA signal of the hydrogel over the time span. i) PA‐B mode overlay image with color coded ROIs and their corresponding spectra in (j) show that ROIs with 730 nm versus 680 nm peak PA signal can localize hydrogel fragments from released MB‐Dox in live tissue. Scale bars: 2 mm. Spikes in PA spectra caused by pulse in live mouse while scanning.

The photoacoustic contrast of the hydrogel was next evaluated in vivo. First, the hydrogel was injected subcutaneously with a 21‐gauge needle into the dorsal site of the mouse. The hydrogel was immediately localizable using PAI, and signal was maintained in the exact same location over the span of a week (**Figure** **5**a–f). The PA spectra was taken at the region of interest (ROI) that surrounded the signal, and the absorbance peak was found to be at 750 nm. The spectrum of this ROI, however, had a gradual blue shift from 750 to 710 nm over that time (Figure 5g). This directly illustrates the gel's degradation and decreased weight percentage.

It was important to assess the MB‐Dox's ability to maintain PA visibility even after its release from the degraded hydrogel. There were several other regions in surrounding tissue that also emitted PA signal, but spectral scanning helped corroborate if those signals were from MB‐Dox or background from endogenous molecules. Spectral scanning successfully localized sites of free MB‐Dox because the spectra for these sites indicated a PA peak at 680 nm, which was identical to the spectral behavior from free MB‐Dox in water (blue, purple, and orange ROIs with corresponding color‐coded spectra in Figure 5i,j). The spectra taken from these regions were compared to control background PA sites, which gave off broad, peak‐free spectra that most likely originated from blood and hemoglobin, such as seen from the pink spectrum in Figure 5j corresponding to the pink outlined ROI in Figure 5i. Other regions of PA intensity maintained a spectral peak at 730 nm that was separate from the originating hydrogel location, which indicated fragments of the hydrogel travelled to other sites in tissue (green ROI and spectra in Figure 5i,j). This study confirms that when the wavelength of the free and loaded drug is established, it can be monitored in real‐time and in live tissue.

**Figure 6:**
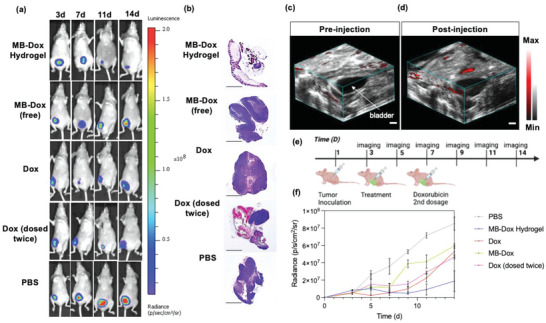
Hydrogel performance for intraperitoneal antitumor efficacy. a) Representative bioluminescent and photograph overlays of mice tumor burden when treated with different formulations over time demonstrate the hydrogel's efficient release and thus administration of doxorubicin. b) H and E images of the harvested peritoneal tissue after the treatment period: Dark purple indicates dense growth of tumor cells (scale bar = 2 mm. c,d) Pre‐ and postinjection of the photoacoustic hydrogel intraperitoneally, next to the bladder of the mouse on its right side Scale bar = 2 mm. e) Timeline of tumor burden study. f) Quantified tumor radiance against time shows mice treated with the MB‐Dox hydrogel showed the most effective anti‐SKOV3 activity (*n* = 5).

The initial 740 nm PA intensity wavelength from the hydrogel becomes increasingly important in vivo as the SBR decreases especially as incident PA wavelengths approach 532 nm.^[^ ^42^ ^]^ The peritoneal cavity, for example, is a common site for a wide class of reproductive and stomach cancers that have developed into advanced stages.^[^ ^43^, ^44^ ^]^ However, its highly dense networks of blood vessels make the region particularly difficult for many PA contrast agents to maintain adequate SBR.^[^ ^45^ ^]^ When the hydrogel was intraperitoneally injected into the mouse (**Figure** **6**c,d and Figure S11, Supporting Information), it was localizable as much of the background was minimized when the mouse was scanned at a 740 nm laser (Figure S11, Supporting Information).

#### 2.3.2 Hydrogel Therapeutic Efficacy In Vivo

The antitumor efficacy of the hydrogel was finally examined. The hydrogel was studied with an SKOV3‐luciferase intraperitoneal murine model, and the tumor burden was monitored by observing changes in bioluminescent signal over 14 days (Figure 6e). The loaded hydrogel's efficacy was compared to the following controls: (1) Free MB‐Dox intraperitoneally injected at the same concentration of loaded MB‐Dox in the hydrogel (9 mg kg^−1^), (2) Dox intraperitoneally injected at the same concentration of the MB‐Dox (9 mg kg^−1^), (3) Dox administered at half of the concentration, but twice throughout the period (4.5 mg kg^−1^), and (4) PBS as the negative control. These controls provide direct comparison between the hydrogel and its counterpart drugs as well as single versus periodic dosing. These different cases were intraperitoneally injected into the mice 3 days after they were inoculated with the SKOV3‐luc cells. Before drug injections, luminescent imaging confirmed that each mouse had the same relative bioluminescence (Figure 6a and Figure S12b, Supporting Information).

Three days after drug injections, the mice injected with single dosage of dox had a direct response as the bioluminescence decreased by 54.2%. Meanwhile, the burden of the hydrogel remained relatively constant, as the bioluminescence did not significantly increase or decrease since it was initially injected. However, 7 days after the dosage, the mice with the hydrogel showed significant decrease in tumor bioluminescence, the lowest signal compared to the other cases. By the end of the study, the hydrogel treatment resulted in 77.91% (+/− 8.79%) decreased radiance, while free MB‐Dox, single and double dosed dox resulted in 30.24% (+/− 5.78%), 38.92% (+/− 10.28%), and 55.67% (+/− 9.17%) decreased radiance, respectively (Figure 6f and Figure S12b, Supporting Information). This trend remained in agreement with histology analysis of the mice subjects ex vivo, which revealed that there was less proliferation of the xenografted cancer cells in the live tissue, and that there was notably increased red fluorescence in the tumor tissue from the hydrogel‐delivered MB‐Dox, as opposed to when the MB‐Dox was administered freely (Figure 6b and Figure S13, Supporting Information). These results demonstrate that when compared to conventional chemotherapy, the hydrogel displays more efficient antitumor efficacy (*p* = 0.0015). This improved antitumor efficacy supports past reports on a hydrogel's advantageous use as a drug delivery vehicle: The slow matrix erosion enables a more gradual release of the drug. This in turn reduces toxic levels of free doxorubicin, avoids a rapid decrease in drug concentration from fast metabolism, and prolongs therapeutic effects over a wider treatment timeline.^[^ ^46^, ^47^, ^48^ ^]^

**Figure 7:**
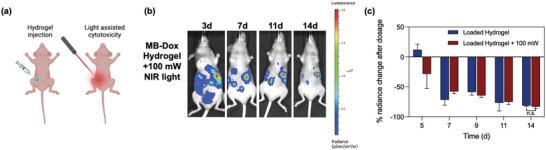
NIR light activated cytotoxicity from the hydrogel. a) Schematic shows setup of exposing the injected hydrogel to a 660 nm laser for NIR‐activated cytotoxicity. b) Bioluminescent images of a mouse subject shows decreased radiance relative to the hydrogel treated mice in Figure 6a. c) Analysis of radiance shows that while light activation allowed an earlier decrease in radiance from the loaded hydrogel, the endpoint therapeutic efficacy was not significantly different (*p* = 0.545, *n* = 5).

Supplementary therapeutic effects from NIR laser activated MB‐Dox remained in question. Therefore, a group of mice injected with the loaded hydrogel were further irradiated with an NIR laser (660 nm, 100 mW) for photoactivation (**Figure** **7**a). Like the in vitro experiments (Figure S6, Supporting Information), the endpoint tumor burden did not lead to a significant decrease in bioluminescence when compared to the loaded hydrogel that was not exposed to the light, as the NIR‐irradiated group led to an average of 80.2% decreased radiance (+/− 5.34%) (*p* = 0.545). Yet interestingly, decreased radiance occurred earlier when compared to the loaded hydrogel alone, indicating the further light‐activated hydrogel led to localized cytotoxicity that in turn amounted to a more expedited tumor toxicity (Figure 7b,c). Because the payload and the hydrogel have demonstrably robust photostability, and because drug release surveillance is more reliant on spectral shifts for quantitative evaluation, both imaging‐assisted monitoring and light‐assisted therapy can occur without significant risk of signal loss.

The DNA hydrogel had three roles: (1) The hydrogel could load a high amount of the MB‐Dox compound due to strong intramolecular affinity between the compound and the DNA framework. (2) The dense intercalation of the highly concentrated compound in the DNA framework promoted J‐aggregation that led to distinct PA spectral shifts for quantitation of drug release. (3) Gradual erosion of the hydrogel due to nuclease activity led to the slow and local release of the MB‐Dox drug so that the antitumor efficacy from the formulation outperformed doxorubicin, despite doxorubicin's lower IC50 value relative to MB‐Dox. This report exemplifies that the formulation of therapeutics alone can offer unique advantages that may enhance the efficacy of the drug itself.

## 3 Conclusion

This work introduced a hydrogel that possesses chemotherapeutic efficacy while offering comprehensive monitoring of drug release and biodistribution via real‐time bioimaging. While this study sought to specifically investigate a DNA hydrogel's ability to load photoacoustic anticancer drugs and control their release through gradual nuclease activity while monitoring the state of the drug, several other accessory features can be considered with this system due to DNA technology's vast multifunctionality. For example, immunotherapeutic CpG sequences and cancer cell targeting aptamer motifs can be embedded in the DNA hydrogel itself. This work illustrates hydrogels as effective means of drug delivery, where the release of a drug is preferably gradual. In the future, hydrogel materials and formulations can be further evaluated for a wide range of treatment applications, such as embedded within bolus for stomach cancers, topically applied for melanoma and other skin‐local cancers, transmucosal administration, or direct injection into neck cancers.

## 4 Experimental Section

### Synthesis and Purification of MB‐Dox

“MB‐Dox” was synthesized using amine linkage of succinimidyl ester (NHS) activated MB with the sugar moiety of doxorubicin. MB‐NHS ester and doxorubicin powders were mixed together with TEA at 1:1:1.5 molar ratios in DMSO, and allowed to react overnight at 300 RPM and 30 °C in the dark. This reaction was then dried under vacuum centrifugation using Vacufuge Plus (Eppendorf, Hamburg) at 60 °C for 2 h.

Crude dry pellets were resuspended in MeCN/H_2_O (30:70 v/v) and purified via isocratic RP‐HPLC with a Shimadzu LC‐40 on a Shim‐pack C18 column (5 µm) over 70 min. During purification, fractions were collected and diluted to 90% (v/v) MeOH before analysis with ESI‐MS in positive mode, centroid scan on Micromass Quattro Ultima Triple Quadrupole mass spectrometer. Pure products were then lyophilized and stored in the dark for later use.

### Optical Characterization of MB‐Dox

Absorbance spectra were measured with a BioTek Synergy H1 plate reader. Samples were measured in 100 µL volumes in 96‐well plates, and absorbance measurements were taken from 400 to 800 nm.

### Nuclear Magnetic Resonance Spectroscopy

Dry MB‐Dox was dissolved in DMSO‐*d*_6_ with a 300 mHz Bruker Avance III HD NMR spectrometer at room temperature. The chemical shifts were calibrated using the nondeuterated DMSO residue (2.50 ppm) in the deuterated solvent as an internal reference from the literature^[^ ^49^ ^]^

### DNA Hydrogel Preparation

DNA strands as described in Table S1 (Supporting Information) were ordered from Integrated DNA Technologies (IDT). Briefly, amplifying strands were heated at 95 °C for 5 min and were then quenched on ice and cooled down to 4 °C. These were then mixed at equal ratios and then 50× higher with the initiating strand at 1XTAEMg Buffer (40 × 10^−3^ m Tris‐base, 20 × 10^−3^ m acetic acid, 2 × 10^−3^ m EDTA, 12 × 10^−3^ m Mg acetate) and left overnight at room temperature.

### Hydrogel Loading

Dry MB‐Dox was first resuspended in pure Milli‐Q water and was pipetted into the DNA hydrogel and the mixture was left to react overnight. The hydrogel was gently lifted from the supernatant of the reactant and was rinsed by resuspending, removing, and resuspending in fresh Milli‐Q water twice. The supernatant was analyzed via absorbance spectroscopy to estimate DLE. Drug loading content was calculated by taking the mass ratio between the loaded MB‐Dox and total loaded hydrogel.

### PA Imaging

PA images of in vitro samples were acquired with a Vevo 2100 LAZR (VisualSonics) using a 21 MHz transducer (LZ‐250). Samples were loaded into 0.86 mm polyethylene tubes and fixed in parallel with a 3D printed sample holder. One tube was filled with reaction solvent (water) to serve as a reference. The fixed samples were placed 1 cm below the transducer in a vessel filled with water. Single wavelength scans were operated at 680 and 730 nm at a frame rate of 20 Hz. For 3D PA images, the transducer was scanned with a stepper motor along the axial dimension of the tubes. PA spectra were taken from 680 to 900 nm with a step size of 2 nm.

### Cell Culture

SKOV3 cells were cultured using McCoy's 5A medium supplemented with 10% fetal bovine serum and 1% penicillin‐streptomycin. Cells were incubated at 37 °C and media was replaced every 1–2 days. Cells were passaged at 75–85% confluence with 0.25% Trypsin EDTA.

### Cytotoxicity Assays

Cells were seeded into 96‐well plates (1100 cells per well) overnight. The media was then replaced with media containing either MB‐Dox, Dox, MB, or the loaded hydrogel at different concentrations for 48 h. For PDT studies, the cells were incubated with MB‐Dox for 2 h before half of the samples were exposed to 100 mW of 660 nm light for 10 min. These cells were left to incubate for 48 h and were compared to those left in the dark. A resazurin assay was used to evaluate the cell viability for all cytotoxicity experiments.

### Animal Studies

All mice studies described below were performed in accordance with National Institutes of Health (NIH) Guidelines approved by the Institutional Animal Care and Use Committee (IACUC) under protocol S15050 at University of California, San Diego. Female J:NU mice of 5 weeks of age were used for all in vivo experiments. For imaging experiments, mice were anesthetized with isoflurane. For both imaging and therapeutic assessment, hydrogels were administered at a 9 mg kg^−1^ dosage of the MB‐Dox compound, which falls under the maximum tolerated dosage (MTD) or MB and doxorubicin (>20 mg kg^−1^ and 10 mg kg^−1^, respectively) in rodents.^[^ ^50^, ^51^ ^]^

### In Vivo PA Imaging of Hydrogel

The hydrogel was injected with a 21‐gauge syringe subcutaneously into the dorsal site of a mouse after resuspension in PBS. The injected region was subsequently imaged on the mouse on days 0, 1, 3, 5, 7, and 8 under the same PA and B‐mode gain. PA spectra were also taken during this time. Photoacoustic images were analyzed on ImageJ to evaluate intensity.

### In Vivo Therapeutic Study

Mice were divided into five groups. 800 000 SKOV3‐luc cells were injected (with 50% Matrigel/PBS v/v) intraperitoneally on the right side of the mice. On day 3, the mice were intraperitoneally injected with MB‐Dox loaded hydrogel (9 mg kg^−1^), Dox (9 mg kg^−1^), MB‐Dox (9 mg kg^−1^), Dox for periodic dosing (4.5 mg kg^−1^), and PBS. On day 7, the mice with periodic dosing were injected with Dox once more (4.5 mg kg^−1^). To measure tumor burden, mice were imaged with D‐luciferin on days 3, 5, 7, 9, 11, 14 with 100 mg kg^−1^ dosage in PBS. The bioluminescence was measured and imaged via IVIS Perkin‐Elmer Illumination and LivingImage software. For laser‐activated toxicity, the mice were exposed to a 100 mW 660 nm laser for 10 min every 2 days.

For ex vivo analysis, tissues from the injection site of the mice were extracted, fixed, and submitted to Pacific Rim Pathology (San Diego) for sectioning and H&E staining.

### Data and Statistical Analysis

All presented data in this manuscript was not preprocessed or normalized. Values and error bars represent mean and standard deviations of measurements between replicates, respectively. Sample sizes are specified in the captions of the figures for each quantitated result. One sided *t*‐tests were used to evaluate differences between groups. For the in vivo therapeutic study, the hydrogel was compared to both the single and double‐dosed doxorubicin cases using ANOVA analysis.

## Conflict of Interest

The authors declare no conflict of interest.

## Supporting information

Supporting Information

## Data Availability

The data that support the findings of this study are available from the corresponding author upon reasonable request.
