# Supplementary material for: Supramolecular Loading of DNA Hydrogels with Dye–Drug Conjugates for Real‐Time Photoacoustic Monitoring of Chemotherapy
Source: Adv Sci (Weinh). 2022 Nov 20;10(1):2204330. doi: 10.1002/advs.202204330 (PMC9811488; doi:10.1002/advs.202204330)
Supplement: Supplementary file 1 — Supporting Information [file ADVS-10-2204330-s001.pdf]

ADVANCED SCIENCE
Open Access

Supporting Information

for *Adv. Sci.*, DOI 10.1002/advs.202204330

Supramolecular Loading of DNA Hydrogels with Dye–Drug Conjugates for Real-Time Photoacoustic Monitoring of Chemotherapy

*Raina M. Borum, Colman Moore, Yash Mantri, Ming Xu and Jesse V. Jokerst**

Supporting Information

Supramolecular Loading of DNA Hydrogels with Dye-Drug conjugates for real time Photoacoustic Monitoring of Chemotherapy

*Raina M. Borum, Colman Moore, Yash Mantri, Ming Xu, and Prof. Jesse V. Jokerst**

Strand name	Sequence (5'→ 3')
HG1	GATCGCGATCCTGGCTCCTGTGATTGTGCTCTAGACATCGCTAGAGCACAATCACAGG
HG2	CTAGAGCACAATCACAGGAGCCAGTTTTCCTGTGATTGTGCTCTAGCGATGT
HGIn	CTA GAG CAC AAT CAC AGG AGC CAG

Table S1. Sequences used for the DNA hydrogel, which was directly from Wang et al's design.[1] 'HG1' and 'HG2' are the amplifying strands that crosslink together after they recognize the initiating 'HGIn' strand that catalyzes the gelation.

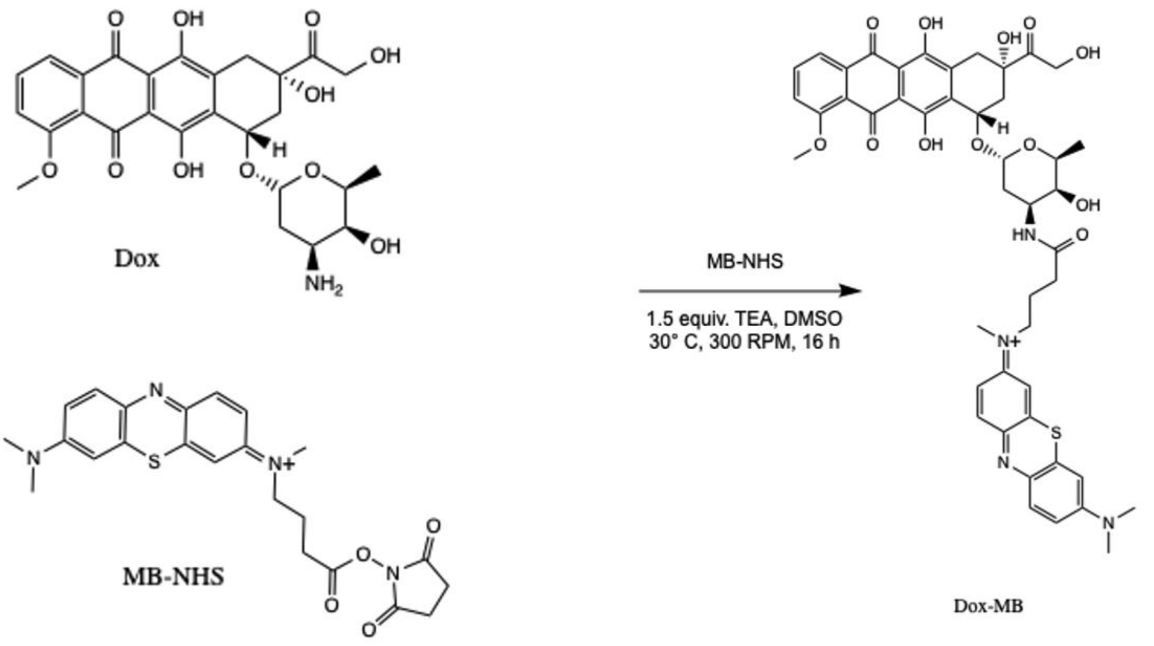

Figure S1. Reaction scheme on the synthesis of MB-Dox. NHS activated Methylene Blue is linked with Doxorubicin through a simple amine reaction between the sugar moiety and NHS tag on doxorubicin and methylene blue, respectively. The process was assisted by triethylamine (TEA) in DMSO over 16 hours.

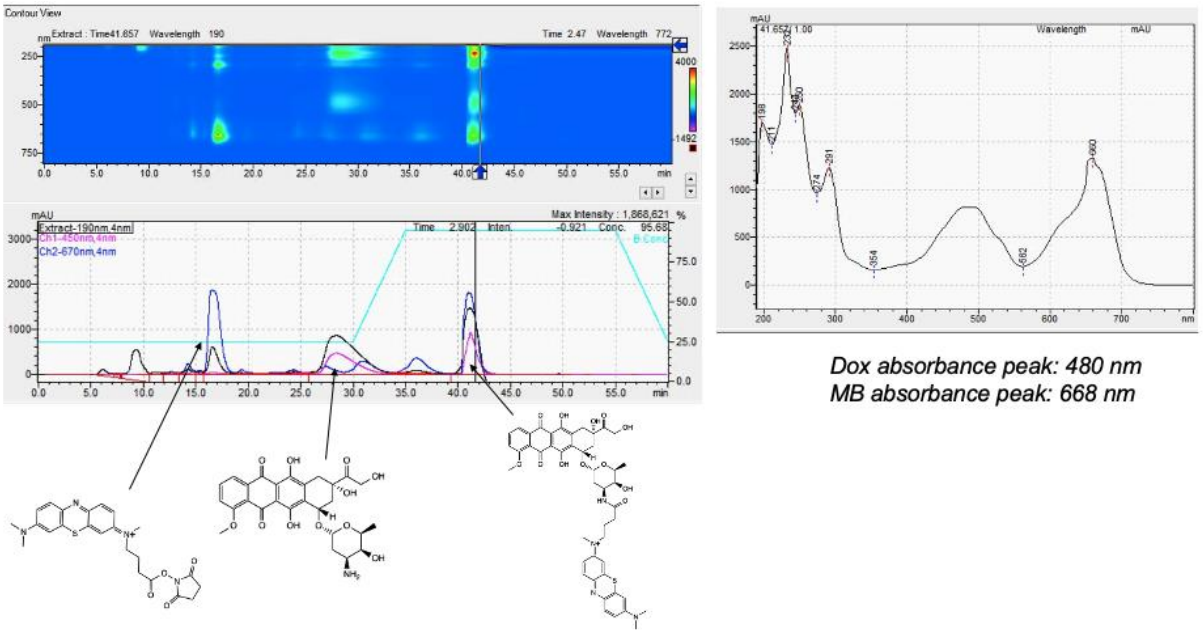

Figure S2. RPP-HPLC liquid chromatogram of the purification process of MB-Dox. Unconjugated MB and Dox fractions were eluted in isocratic 30% MeCN/H2O (v/v) between 15 and 30 minutes, respectively, while the product was released at 90% MeCN (B) concentration. The same protocol was run to validate pure MB-Dox products as in Figure 2B in the main text.

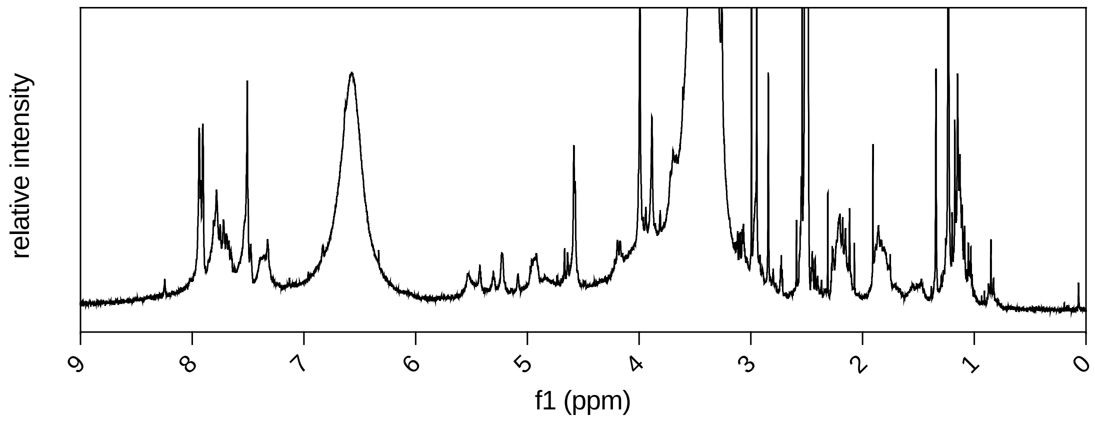

Figure S3 ^{1}H NMR (300 MHz, DMSO-d_6, δ)

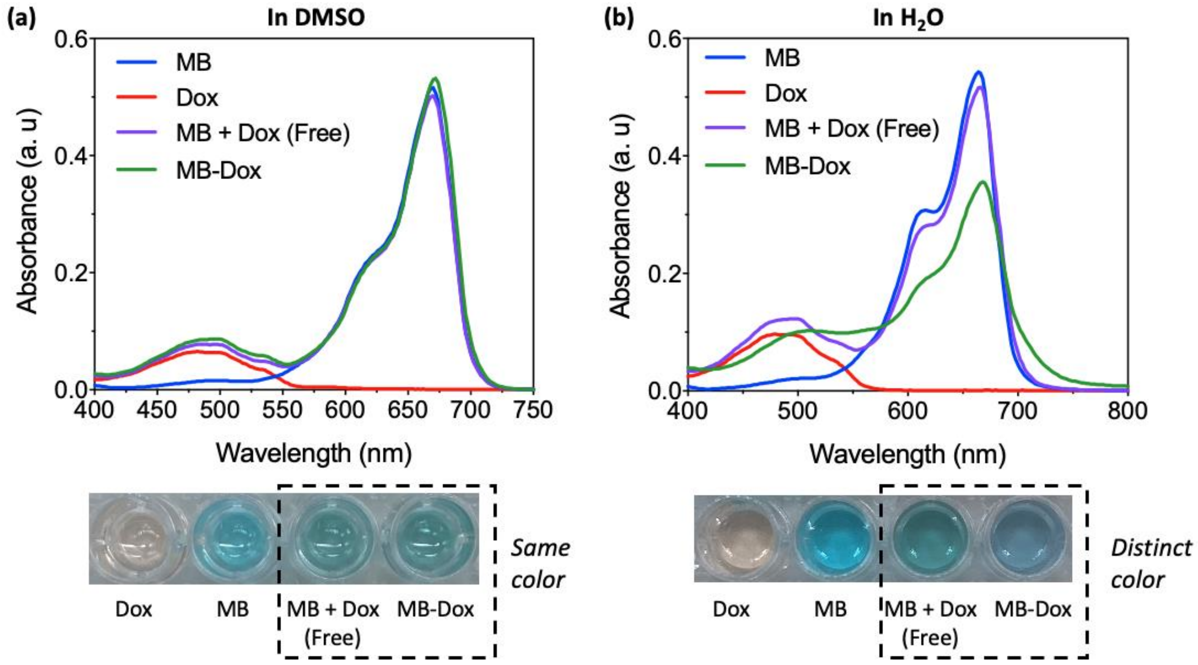

Figure S4 Difference in optical absorbance spectra in MB-Dox when in DMSO versus water. (a) in DMSO, the absorbance peak of MB-Dox is nearly identical to additive curves MB and Dox when mixed together. However, when observed in water (b) the MB-Dox compound exhibited a slight 3 nm red shift.

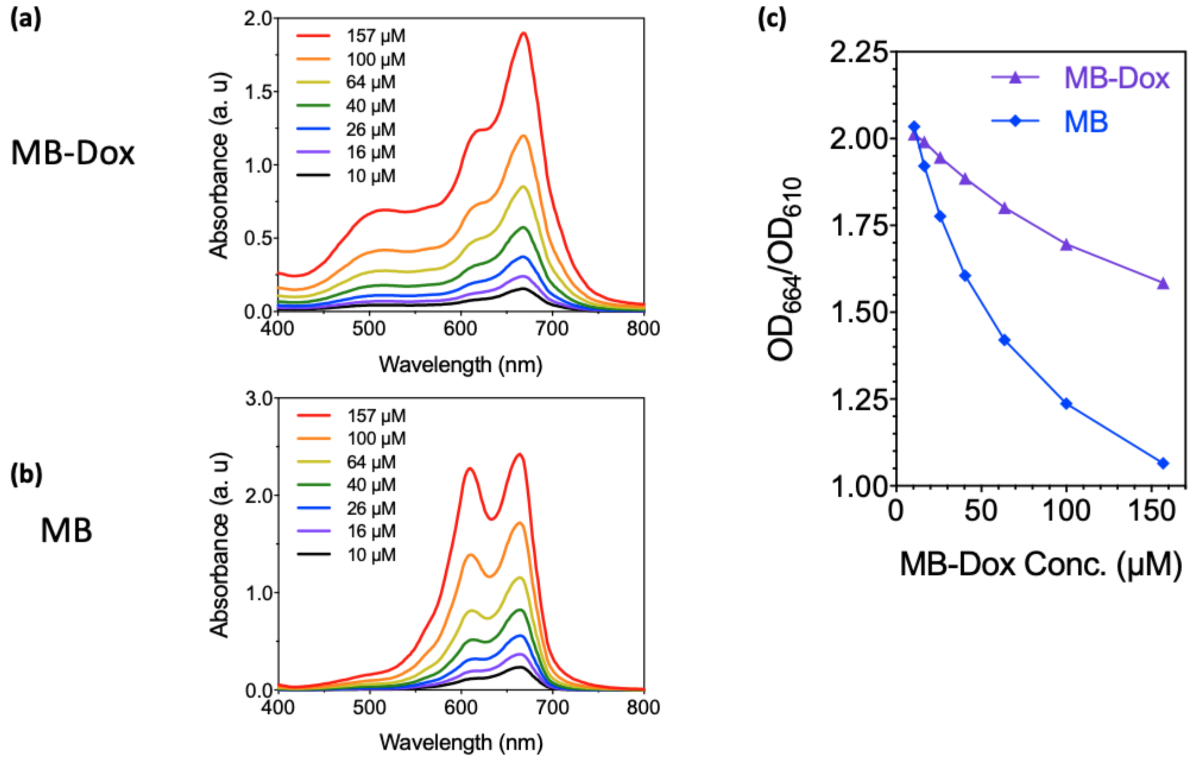

Figure S5 Optical comparison between MB-Dox and MB. (a) When MB-Dox has increasing micromolar concentrations in water, there is no increase in a secondary 610 nm peak that is usually seen in MB dye alone, which is typically caused by dimerization between the dye molecules. (c) Ratiometric absorbance measurements between MB-Dox and MB at increasing concentrations further demonstrate MB-Dox's structural protection over self-dimerization in water.

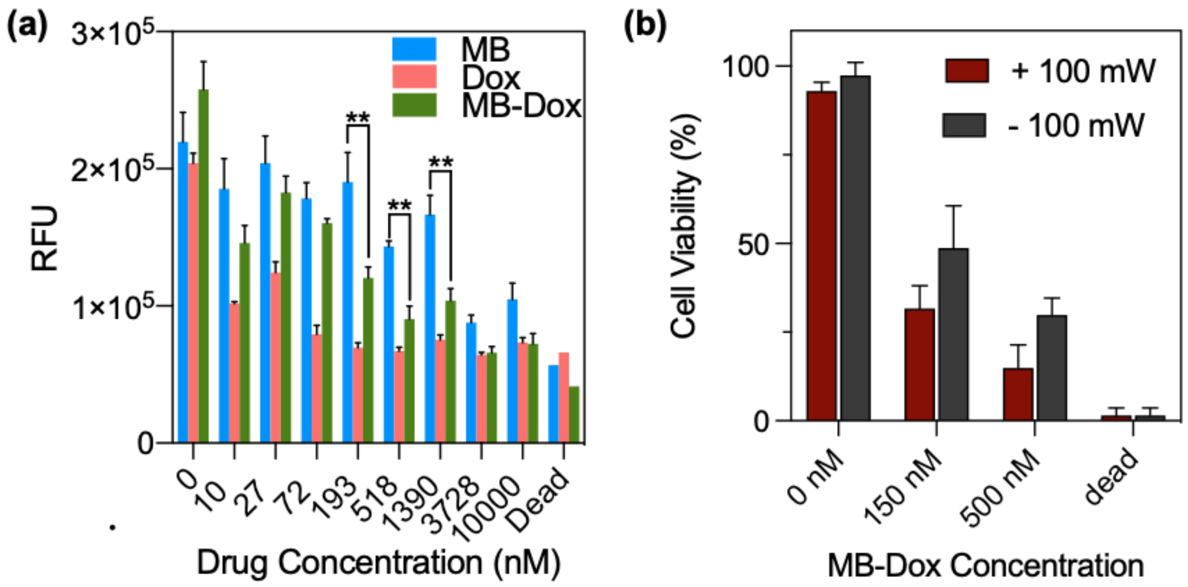

Figure S6 MB-Dox cytotoxicity profile via resazurin assay, where increased fluorescence indicates less toxicity against the growing SKOV-3 cells. (a) Cytotoxicity of MB-Dox, MB, and Dox against SKOV-3 cells (**$P<0.001$, student's t-test, n=3). (b) Photodynamic therapy demonstration shows higher cytotoxicity when cells incubated with MB-Dox are further exposed to 100 mW red light (n=3).

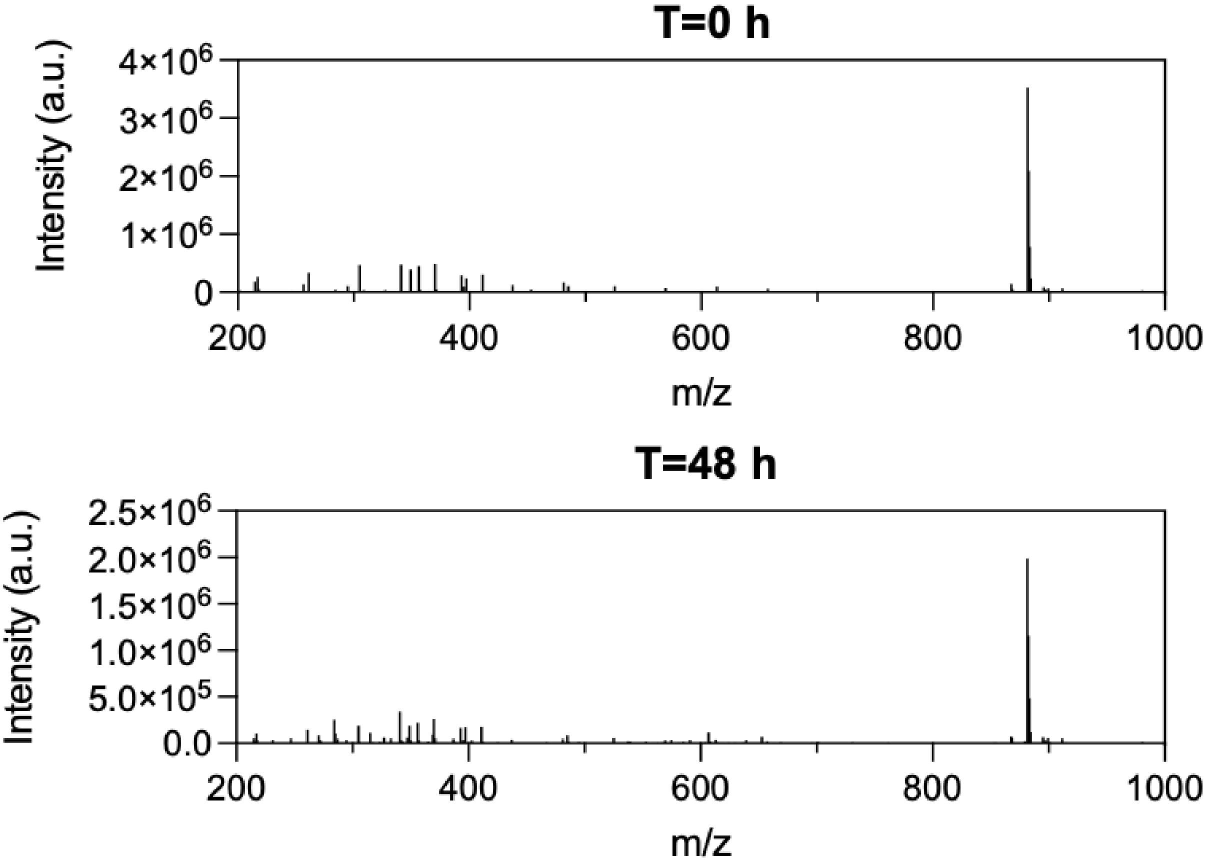

Figure S7 Molecular stability of MB-Dox in cell culture media at 37°C over 48 hours as confirmed via ESI-MS.

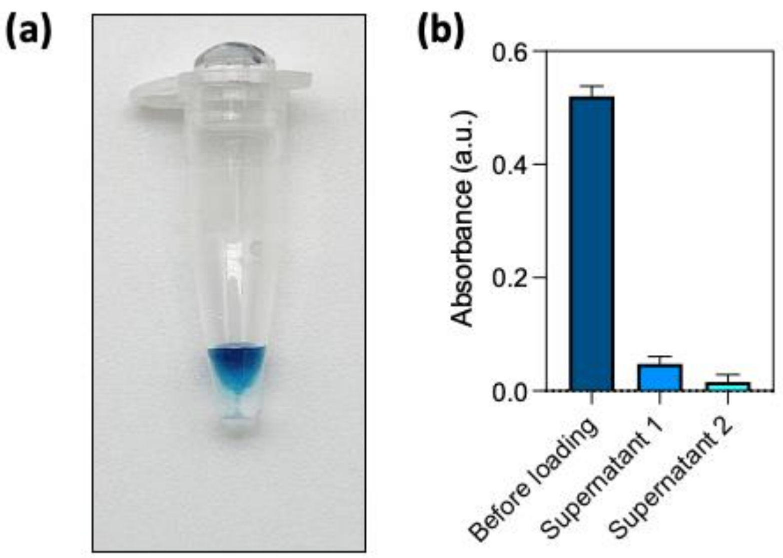

Figure S8 Hydrogel loading efficiency with MB-Dox (a) photograph of hydrogel floating in nuclease-free water of centrifuge tube shows that the MB-Dox does not leak out of the hydrogel and into the supernatant. (b) Quantified optical density of supernatants after rinsing loaded hydrogel demonstrate a 91.29% loading efficacy (n=3).

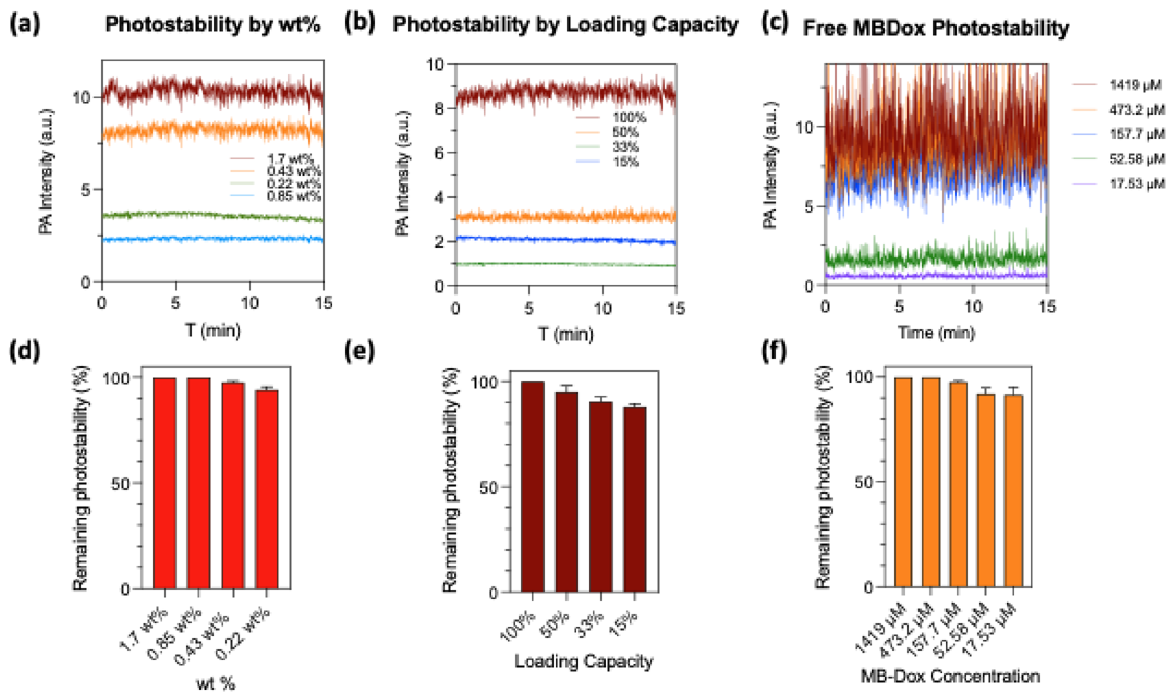

Figure S9 Photostability of loaded hydrogels and MB-Dox. (a)-(c) show raw data of photoacoustic intensity during 15-minute constant excitation for hydrogels based on wt%, loading capacity, and free MB-Dox in water, respectively. Panels (d)-(f) of corresponding decrease in PA intensity from panels (a)-(c) show that photostability does drop below 87%.

The concentrations for free MB-Dox were chosen for photostability testing because these fit within the typical concentration of MB-Dox loaded in the hydrogel. (n=3)

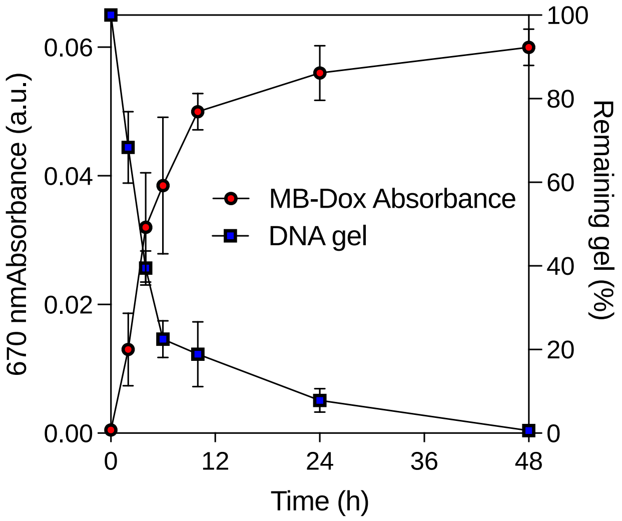

Figure S10 Hydrogel degradation and MB-Dox release profile via optical absorbance measurements of a 0.22 wt% loaded hydrogel (n=3).

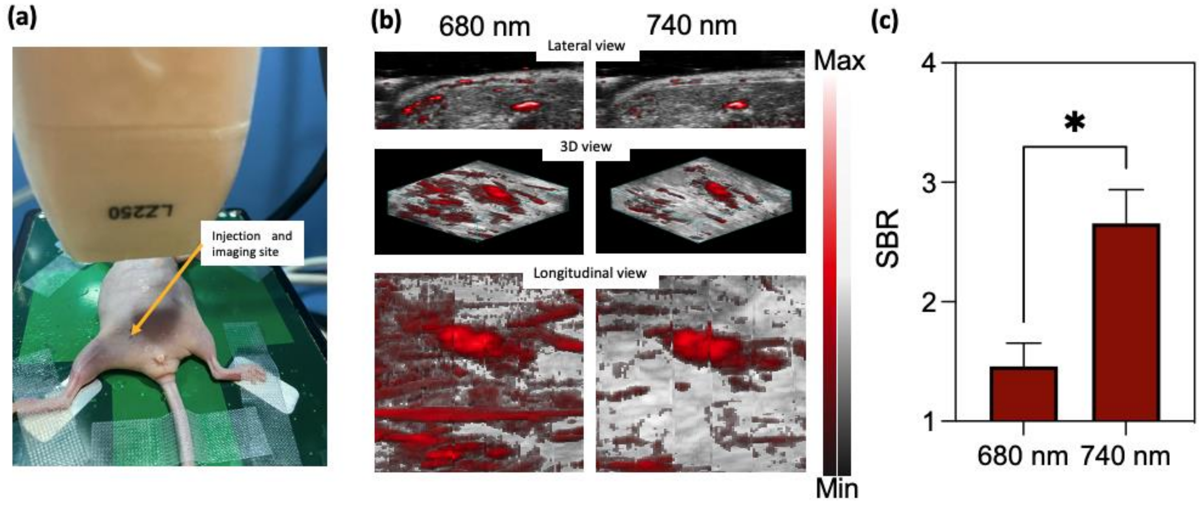

Figure S11 Intraperitoneal PA imaging in vivo (a) setup for in vivo intraperitoneal imaging, where the transducer is above the injection site of the mouse while it is fixed on a heating pad and anesthetized. (b) lateral, 3D, and longitudinal views of the photoacoustic hydrogel after intraperitoneal injection shows significantly improved contrast from the gel relative to endogenous PA by changing the NIR wavelength from 680 nm to 740 nm. (c) Although the same gel was imaged under both wavelengths, the 740 nm wavelength scanning showed a

significantly (p=0.0129, student's t-test) enhanced signal to background ratio (SBR) over 680 nm scanning.

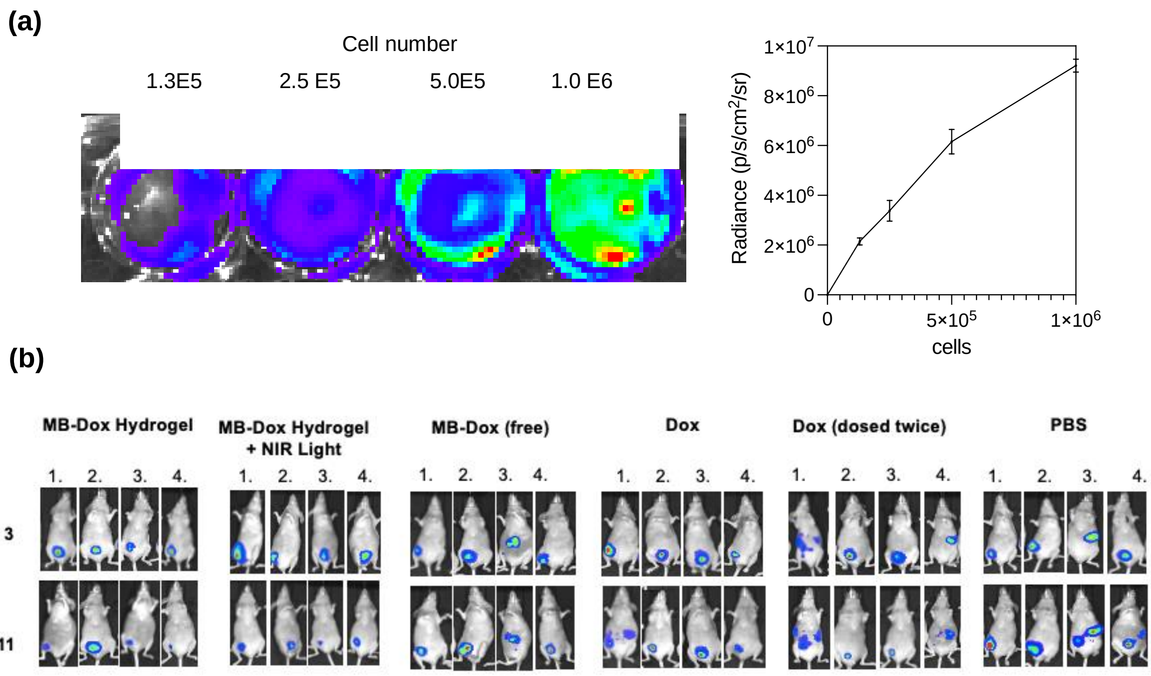

Figure S12 (a) in vitro image and quantified bioluminescence of luciferase expressive SKOV-3 cells. (n=3) **(b)** Anti-tumor efficacy via in vivo bioluminescence monitoring: Bioluminescent images of the remaining four of five mice per group show similar relative bioluminescence three days after tumor inoculation, while the mice treated with the loaded hydrogel showed the most decreased bioluminescence from the gradual release of the MB-Dox payload.

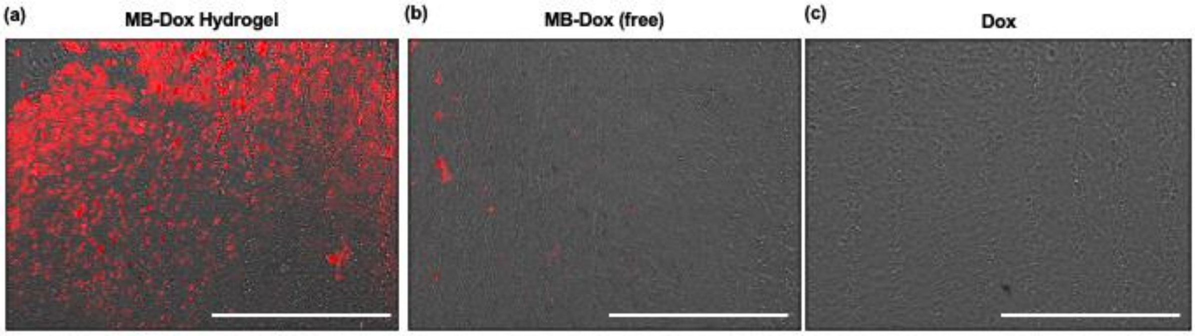

Figure S13 Ex vivo fluorescent histology analysis of the harvested peritoneal tissue shows (a) heightened fluorescence inside the cells from accumulated Methylene Blue signal from when the mouse was treated with the loaded MB-Dox hydrogel; the local delivery of the compound from the hydrogel erosion after installation outperforms the delivery of the MB-Dox when it was freely injected while in PBS, as is seen with significantly decreased fluorescence in panel (b). As a control, the mouse treated with free dox did not show any fluorescence as seen in (c). Scale bar=100 micron.

Reference

[1] J. Wang, J. Chao, H. Liu, S. Su, L. Wang, W. Huang, I. Willner, C. Fan, *Angew. Chem. Int. Ed.* **2017**, *56*, 2171.
